# Feasibility and accuracy of point-of-care pocket-size ultrasonography performed by medical students

**DOI:** 10.1186/1472-6920-14-156

**Published:** 2014-07-28

**Authors:** Garrett Newton Andersen, Annja Viset, Ole Christian Mjølstad, Øyvind Salvesen, Håvard Dalen, Bjørn Olav Haugen

**Affiliations:** 1 MI Lab and Department of Circulation and Medical Imaging, Norwegian University of Science and Technology, Postboks 8905, 7491 Trondheim, Norway; 2 Clinic of Cardiology, St. Olav Trondheim University Hospital, Trondheim, Norway; 3 Levanger Hospital, Nord-Trøndelag Health Trust, 7600 Levanger, Norway; 4 Clinic of Radiology and Nuclear Medicine, St. Olav Trondheim University Hospital, Trondheim, Norway; 5 Department of Cancer Research and Molecular Medicine, Norwegian University of Science and Technology, Trondheim, Norway

**Keywords:** Echocardiography, Point-of-care ultrasound, Bedside, Medical student, Hand-held

## Abstract

**Background:**

Point-of-care ultrasound performed by clinicians is a useful supplement in the treatment and assessment of patients. We aimed to investigate whether medical students with minimal training were able to successfully acquire and interpret ultrasound images using a pocket-size imaging device (PSID) as a supplement to their clinical practice.

**Methods:**

Thirty 5th year (of six) medical students volunteered to participate. They were each given a personal PSID device to use as a supplement to their physical examination during their allocated hospital terms. Prior to clinical placement the students were given three evenings of hands-on training with PSID by a board certified radiologist/cardiologist, including three short lectures (<20 min). The students were shown basic ultrasound techniques and taught to assess for basic, clinically relevant pathology. They were specifically instructed to assess for the presence or absence of reduced left ventricular function (assessed as mitral annular excursion < 10 mm), pericardial effusion, pleural effusion, lung comets, hydronephrosis, bladder distension, gallstones, abdominal free-fluid, cholecystitis, and estimate the diameter of abdominal aorta and inferior vena cava.

**Results:**

A total of 211 patients were examined creating 1151 ultrasound recordings. Acceptable organ presentation was 73.8% (95% CI 63.1-82.6) for cardiovascular and 88.4% (95% CI: 80.6-93.6) for radiological structures. Diagnostic accuracy was 93.5% (95% CI: 89.0-96.2) and 93.2% (95% CI: 87.4-96.5) respectively.

**Conclusion:**

Medical students with minimal training were able to use PSID as a supplement to standard physical examination and successfully acquire acceptable relevant organ recordings for presentation and correctly interpret these with great accuracy.

## Background

We are increasingly reliant upon expensive and time-consuming biochemical and radiologic diagnostics to aid us in our evaluation of patients. Unfortunately this still results in major diagnostic errors in up to 30% of patients at autopsy [1-3]. Furthermore the increasing age and chronicity of the western population highlights the need for improved out of hospital diagnosis and treatment.

Point-of-care ultrasonography allows for the near instantaneous acquisition of real-time dynamic images, which can be correlated directly to the patient’s signs and symptoms [4,5]. It has been shown to increase diagnostic accuracy, rapidly and cost effectively in the hands of experts and non-experts [6-13]. Furthermore, portable ultrasonography is a valuable teaching tool in medical anatomy and physiology as well as physical examination [14-18]. Despite this most medical students are not routinely educated in the clinical use of point-of-care ultrasonography, as they are in more widely accepted and traditional techniques, such as the stethoscope. This may in part be due to the lack of evidence regarding the bedside use of pocket-size ultrasound by medical students.

We aimed to investigate whether medical students with minimal training were able to successfully acquire and interpret ultrasound images using a pocket-size imaging device (PSID) as a supplement to their clinical practice.

## Methods

### Medical students

The fifth year (of six) medical students eligible to participate in the study based on planned hospital rotations received verbal and written information from the authors regarding the study. Participation in the study was not part of the students’ curriculum and all participating students were volunteers. The first 30 students whom volunteered were included in the study. There were no further inclusion or exclusion criteria. The number of participating students was limited to the number of available PSID. The medical students had similar limited experience in ultrasound.

### Study population

All patients over 18 years of age, encountered in-hospital and at outpatient clinics during the students’ clinical placement periods were eligible for inclusion. The patients were included from a total of seven regional hospitals between January-May 2012. There were no exclusion criteria, and all participating patients provided informed consent.

### Training and education of medical students

The medical students received three evenings (nine hours) of combined theoretical and practical training in the use and interpretation of ultrasound images. The theoretical training was given as short didactical lectures by relevant specialists (cardiologists and radiologists) and focused on basic ultrasound physiology, anatomy and examples of normal and pathological ultrasound images. Students were specifically trained to assess for pathology relevant in the immediate emergency care of patients. They were instructed to assess for reduced left ventricular (LV) function defined as mitral annular excursion (MAE) < 10 mm [19-21], pericardial effusion, pleural effusion, lung comets, inferior vena cava (IVC) diameter and variation, hydronephrosis, bladder distension, gallstones, signs of cholecystitis, diameter of abdominal aorta (AA) and abdominal free-fluid. Practical hands-on training was given by relevant specialists and senior registrars, with students using their personal PSID. Students were encouraged to perform at least 75 examinations prior to placement.

Written informed consent was obtained from all patients. The Regional Committee for Medical and Health Research Ethics had no objections to the study’s conduction, which was conducted according to the Declaration of Helsinki.

### Pocket-size ultrasound examination

The ultrasound examination was performed bedside with a PSID, Vscan (GE Vingmed Ultrasound, Horten, Norway). The device measures 135 × 73 × 28 mm and weighs 390 g, including the phased-arrayed probe. Two-dimensional grey scale and live colour Doppler imaging are offered. The image sector for echocardiographic imaging is 75°. The bandwidth ranges from 1.7 to 3.8 MHz and is automatically adjusted. Storage and looping of a cardiac cycle are possible without ECG signal and looping of other structures is predefined and limited to 2 seconds. The device has separate modes optimized for cardiac and abdominal examinations. All images and recordings were saved on the device’s micro-SD card and later transferred to a computer by commercial software (Gateway; GE Vingmed Ultrasound).

The bedside (point-of-care) cardiovascular ultrasound examination was performed with patients in the left-lateral decubitus and/or supine position. Assessment of LV global function was done from the apical four-chamber view using MAE, where MAE < 10 mm was classified as decreased LV function. Pericardial effusions were classified as present or not. The AA and IVC were assessed from the subcostal position. The AA diameter was assessed proximally to the bifurcation and if exceeding 35 mm classified as an abdominal aortic aneurysm (AAA). IVC diameter was measured end-expiratory within two cm from the right atrium orifice. All measurements of dimensions were done on the PSID. With patients in a supine or upright position, the pleura was assessed from left and right thoracic dorsolateral views, and assessed for the presence of pleural effusions and comet tails.

Other abdominal structures and spaces were assessed from a supine position looking specifically for hydronephrosis, bladder distension, gallstones, and signs of cholecystitis, and abdominal free-fluid.

### Accuracy

The students were required to hand-in a log of selected examinations including their own set diagnosis based upon PSID examination. The specialists, one board certified radiologist and 2 board certified cardiologists with special interest in ultrasonography and echocardiography, were asked to categorize the image acquisition of relevant organ as acceptable or unacceptable for clinical interpretation and then determine whether the set diagnosis of the acceptable images were correct or incorrect. The specialists were not blinded to the set diagnosis.

### Statistics

Data not following a normal distribution were presented as median and (interquartile) range. For sufficiently large samples logistic mixed model with random intercepts for students was used to examine estimate proportions. Clopper-Pearson estimates were used for small sample analyses. Sensitivity and specificity, negative and positive predictive value calculations were performed using relevant specialists as “gold standard”.

All the statistical analyses were performed using SPSS for Windows/Mac (version 20.0, SPSS, Inc.) or R version 2.13.1.

## Results

**Figure 1:**
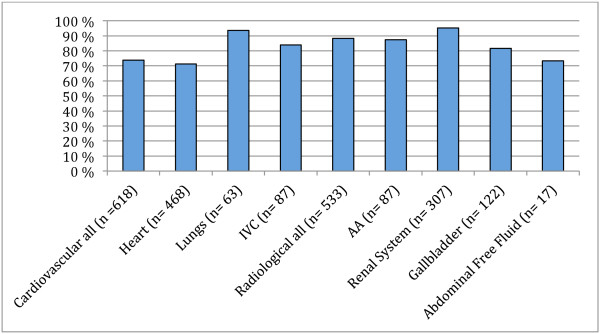
**Acceptable organ presentation.** Cardiovascular all; heart, IVC and Lungs, IVC; Inferior vena cava, Radiological all; includes AA, Renal system, Gallbladder and Abdominal free fluid. AA; Abdominal aorta.

**Figure 2:**
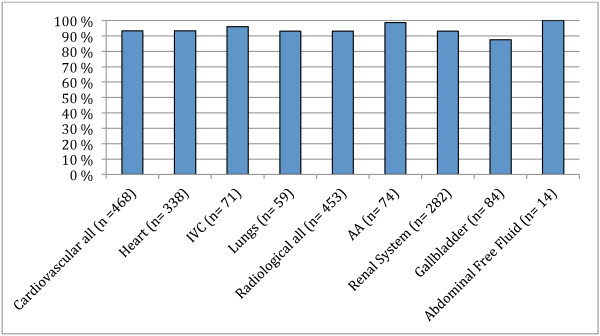
**Correct diagnosis.** Cardiovascular all; heart, IVC and Lungs, IVC; Inferior vena cava, Radiological all; includes AA, Renal system, Gallbladder and Abdominal free fluid. AA; Abdominal ao.

Thirty 5th year (of six) medical students volunteered to participate in the study. At the end of the study period and their clinical placement, 21 (70%) medical students had performed exams using PSID and recorded their results. A total of 211 patients were examined (43% male, 38% female and 18% unrecorded sex), creating 1151 ultrasound recordings. Each student examined a median of 9 (±8, range 1–27) patients, producing a median of 49 (±49, range 5–169) ultrasound recordings. Acceptable organ presentation (Figure 1) was estimated to 73.8% (95% CI 63.1-82.6) for cardiovascular (heart, lungs and IVC) and 88.4% (95% CI: 80.6-93.6) for radiological (AA, renal system, gallbladder and abdominal free fluid) structures. Specifically, students performed best when acquiring images of the lungs and renal system (>93% (95% CI: 84.3-98.2) and found it most difficult to acquire acceptable images of the heart (71.2% (95% CI: 58.7-81.5)) and free fluid (73.2% (95% CI: 41.4-92.7)). The other categories (AA, IVC and gallbladder) had acceptable presentation in >80% (95% CI: 65.2-92.9) of cases. Diagnostic accuracy (Figure 2) was estimated at 93.5% (95% CI: 89.0-96.2) for cardiovascular structures and 93.2% (95% CI: 87.4-96.5) for radiological structures. The specific diagnostic accuracy was on a whole excellent. Diagnostic accuracy was close to 100% for AA (98.6% (95% CI: 92.7-100)) and free abdominal fluid (100% (95% CI: 76.8-100)) and lowest for gallbladder at 87.6% (95% CI: 73.7-95.1). The remaining categories showed diagnostic accuracy > 93% (95% CI: 83.3-99).

The estimated values for sensitivity, specificity, negative and positive predictive values of PSID are presented in Table 1.

**Table 1:** Sensitivity, specificity, positive and negative predictive values

**Pathology to detect**	**N Pathology (N total)**	**Sensitivity % (95% CI)**	**Specificity % (95% CI)**	**PPV % (95% CI)**	**NPV % (95% CI)**
All cardiovascular	156 (468)	95.5 (90.9-97.9)	92.4 (83.7-96.9)	87.0 (75.3-93.4)	97.6 (95.0-98.8)
Cardiac only	115 (338)	98.3 (93.9-100) *	90.8 (78.8-96.7)	84.5 (62.6-95.6)	99.0 (96.4-99.9) *
IVC	20 (71)	84.5 (57.2-96.3)	100 (93.0-100) *	100 (80.5-100)*	94.8 (82.9-98.7)
Lungs	21 (59)	90.5 (68.8-97.6)	94.7 (82.2-99.4)*	90.5 (69.6-98.6) *	94.7 (82.2-99.2)*
All abdominal	104 (453)	92.6 (83–97.1)	92.2 (82.9-96.9)	80.1 (63.3-91.0)	97.5 (92.6-99.2)
AA	12 (74)	91.7 (61.5-98.6)*	100 (94.2-100)*	100 (71.3-100)*	98.4 (91.5-99.6)*
Renal system	48 (282)	89.9 (77.2-95.9)	93.3 (82.5-98.0)	73.1 (48.4-89.6)	97.5 (85.7-99.7)
Gallbladder	35 (84)	94.3 (80.8-99.1)*	85.6 (71.5-93.4)	82.4 (63.7-93.1)	95.5 (84.5-99.3*
Abdominal free fluid	9 (14)	100 (66.2-100)*	100 (48.0-100)*	100 (66.2-100)*	100 (48.0-100)*

*N*; number, *CI*; confidence interval, *PPV* positive predictive value, *NPV*; negative predictive value, *IVC*; inferior vena cava, *AA*; abdominal Aorta. * Clopper-Pearson CI.

## Discussion

Medical students, with a limited amount of training, successfully incorporated the use of point-of-care ultrasonography in their clinical placements. They were able to correctly acquire bedside ultrasound images of cardiovascular and radiological structures in 74 and 88% of their patients and correctly interpret these images in 93% of cases.

An attempt to simulate real life scenarios was done when determining the feasibility and accuracy. In our experience, when non-experts use pocket-size ultrasound at the patients point-of-care they may have the need to clarify or present their ultrasound findings to a specialist for review or guidance. The specialists were in this setting used as the gold standard with regards to statistical analysis and were not blinded to the set diagnosis. Optimally this would have been done by higher order, formal imaging, but that was beyond the scope of this study in terms of logistics and economy.

Other studies have shown that medical students are able to quickly acquire ultrasound recordings of good quality on normal test subjects, in optimal conditions with a standard ultrasound machine and PSID after a brief period of training [22,23]. For the assessment of diagnostic accuracy in our study, only acceptable organ images were used. This may have diluted the true diagnostic accuracy to some extent. However the lack of a formal gold standard/reference made the basis for this, as assessing accuracy in non-acceptable images is useless when no reference is available.

A recent, though smaller study with five final year medical students has shown encouraging results using pocket-size cardiac ultrasonography as an adjunct to standard physical examination in cardiology patients [9]. We have broadened the field, looking at several different organ systems and included diverse groups of hospital and emergency room patients.

The European Association of Echocardiography published a position statement in 2010 regarding with the use of PSID [24]. It supports the use of PSID as a teaching tool in medical schools, as a tool for a fast initial screening in the emergency setting, and as a complement to the standard physical examination.

Previous studies have shown increased accuracy, efficacy and diagnostic impact of pocket-size point-of-care ultrasonography in the hands of experts versus non-experts [6-8,11,13,25]. Thus the benefits of bedside PSID exams increase with increasing proficiency in its use and proficiency has been shown to increase with increasing use [23]. Additionally, ultrasonography has been shown to increase the skills of medical students in core subjects such as anatomy, physiology, and physical examination [9,14-18]. Therefore standardized training with an appropriate education program in the routine use PSID as an adjunct to standard physical examination should start as early as possible in a physician’s career.

### Limitations

The main limitation of this study is the inability to exclude for selection bias. With the use of their logbooks, students were able to select which ultrasound loops were eligible for review. This selection and spectrum bias may have lead to some overestimation of the results for feasibility and accuracy, however the degree of selection bias is in line with similar studies involving unselected residents and nurses [26,27]. Furthermore one student did not hand in a completed logbook and a further eight students did not perform any examinations with PSID and were therefore excluded from the study. The number of students not performing any examinations was probably influenced by several factors. Firstly the use of PSID in their clinical placement was not a mandatory exercise for the medical students. Secondly, as this was a trial of the use of PSID the students received specific instructions not to let the trial come in the way of their other academic responsibilities. Thirdly, the inclusion of patients was performed by the medical students themselves, which may have created a further barrier for its use. Lastly the use of ultrasound imaging is operator dependant, enthusiastic students will likely acquire more and better images reflecting a more realistic picture of it’s clinical use, i.e. those skilled in ultrasound will also be the ones using it the most.

## Conclusion

Medical students with minimal training were able to use PSID as a supplement to standard physical examination and successfully acquire acceptable relevant organ images for presentation and correctly interpret these with great accuracy. Incorporating training of point-of-care ultrasound in medical student education may be one step further towards a more widespread use of ultrasound and a faster and more accurate diagnosis for patients.

## Abbreviations

PSID: Pocket-size imaging device; LV: Left ventricular; MAE: Mitral annular excursion; IVC: Inferior vena cava; AA: Abdominal aorta; AAA: Abdominal aortic aneurysm.

## Competing interests

GE Healthcare provided 20 of the 30 PSID devices used during this study as a loan. No financial support was received.

## Authors’ contributions

GNA participated in the study design and coordination, education of medical students, data processing, performed the statistical analysis and drafted the manuscript. AV participated in the coordination of the study, education of the medical students and review of relevant images. OCM participated in the coordination of the study, education of the medical students and review of relevant images. ØS participated in the design of the study and performed the statistical analysis. HD participated in the design of the study, performed the statistical analysis and helped to draft the manuscript. BOH conceived of the study and participated in its design and coordination, education of the medical students and review of relevant images and helped to draft the manuscript. All authors read and approved the final manuscript.

## Pre-publication history

The pre-publication history for this paper can be accessed here:

http://www.biomedcentral.com/1472-6920/14/156/prepub
